# Real-World Data and Clinical Implications of Next-Generation Sequencing (NGS)-Based Analysis in Metastatic Breast Cancer Patients

**DOI:** 10.3390/ijms25052490

**Published:** 2024-02-20

**Authors:** Fabio Canino, Antonio Tornincasa, Stefania Bettelli, Samantha Manfredini, Monica Barbolini, Luca Moscetti, Claudia Omarini, Angela Toss, Fabio Tamburrano, Giuseppina Antonelli, Federica Baglio, Lorenzo Belluzzi, Giulio Martinelli, Salvatore Natalizio, Ornella Ponzoni, Massimo Dominici, Federico Piacentini

**Affiliations:** 1 Division of Medical Oncology, Department of Medical and Surgical Sciences for Children and Adults, University Hospital of Modena, 41124 Modena, Italy; barbolini.monica@gmail.com (M.B.); angela.toss@unimore.it (A.T.); fabio.tamburrano@unimore.it (F.T.); giusina.antonelli@gmail.com (G.A.); federica.baglio@hotmail.it (F.B.); l.belluzzi@hotmail.it (L.B.); giuliomartin@gmail.com (G.M.); salvatore.natalizio89@gmail.com (S.N.); orny.ponzoni@gmail.com (O.P.); massimo.dominici@unimore.it (M.D.); federico.piacentini@unimore.it (F.P.); 2 Gruppo Oncologico Italiano di Ricerca Clinica (GOIRC), 43126 Parma, Italy; moscetti.luca@aou.mo.it (L.M.); omarini.claudia@aou.mo.it (C.O.); 3 Unità Operativa di Oncologia, ASL I dell’Umbria, 06012 Città di Castello, Italy; antonio.tornincasa1988@gmail.com; 4 Molecular Pathology and Predictive Medicine, Azienda Ospedaliero, Universitaria Policlinico di Modena, 41124 Modena, Italy; bettelli.stefania@aou.mo.it (S.B.); samantha.manfredini@hotmail.com (S.M.); 5 Division of Medical Oncology, Department of Oncology and Ematology, Azienda Ospedaliero, Universitaria Policlinico di Modena, 41124 Modena, Italy

**Keywords:** advanced breast cancer, next-generation sequencing (NGS), target therapy, actionable mutations, tumor mutational burden

## Abstract

Over the last two decades, the use of Next-Generation Sequencing (NGS) in medical oncology has increased the likelihood of identifying druggable mutations that may be potentially susceptible to targeted treatments. The European Society for Medical Oncology (ESMO) currently does not recommend the use of the NGS test to determine the therapeutic course of patients with metastatic breast cancer (mBC) in daily clinical practice. However, the aim of this work is to evaluate the potential contribution of the NGS test in selecting targeted therapies for patients with mBC. Data were retrospectively collected from 101 patients diagnosed with metastatic breast cancer and treated at the Modena Cancer Center between January 2015 and April 2022. A NGS test was performed on the tumor tissue of each patient at the Laboratory of Molecular Pathology of the University Hospital of Modena. This study analyzed the clinical–pathological characteristics and mutational profile of the population using NGS tests, with a focus on actionable mutations that could be targeted in advanced stages of clinical development. The indicator of this study was to quantify the actionable mutations that resulted in a change of cancer treatment. In total, 101 patients with metastatic breast cancer were analyzed, including 86 with luminal phenotype, 10 who were HER2-positive and 5 who were triple-negative. Median age was 52 years. NGS analysis was conducted on 47 samples of primary breast cancer, 52 on metastatic sites of disease and 2 on liquid biopsies. A total of 85 gene mutations were found. The most common mutations were identified in the *PIK3CA* (47%), *FGFR* (19%) and *ERBB2* genes (12%), and to a lesser extent in other genes. Of the 61 patients with pathogenic mutations, 46 (75%) had at least one actionable mutation. Of these, nine received treatment with a molecular target drug: eight patients with a mutation of the *PIK3CA* gene were treated with alpelisib and fulvestrant; one patient with *FGFR1/2* amplifications received TAS120. Median PFS for these patients was 3.8 months. The study results show that using the NGS test on cancer tissue of metastatic breast cancer could influence the therapeutic choices, considering the small sample size and limited follow-up. About 9% of the study population had their therapy modified based on the results of NGS. The growing number of detectable mutations and increased accessibility of the test may lead to a greater number of potential therapeutic implications for the NGS assay. Perspectives suggest that NGS analysis can be implemented in daily clinical practice, particularly in contexts where a Molecular Tumor Board (MTB) is active.

## 1. Introduction

In recent years, advances in diagnostic techniques and pharmacological options have enabled a move towards personalized medicine. This involves analyzing the specific molecular profile of a patient’s cancer to administer druggable agents that target selected gene mutations. Modern methods of DNA sequencing are globally defined Next-Generation Sequencing (NGS), platforms of gene panels that allow many samples to be sequenced simultaneously in a relatively shorter time, with a more affordable cost and higher throughput compared to previous sequencing techniques such as Sanger sequencing. NGS can provide a comprehensive molecular profile of the tumor by sequencing the entire genome to identify various types of alterations that may affect genes and transcripts. To obtain this information, various samples such as biopsies on primary tumor tissue, metastatic sites affecting different organs, and liquid biopsies can be subjected to NGS. The use of NGS can not only guide the clinician in the choice of a possible therapy directed at a specific molecular target, but can also reveal tumor biology and mechanisms of resistance to systemic therapy. However, the analysis of such samples at a molecular level generates a vast amount of data that can be challenging to manage, analyze and interpret. The first step involves collecting and aligning the raw data to a reference genome. The next step is to extract clinically relevant information and translate it to the clinical level. This process requires specific skills and technologies. The use of Next-Generation Sequencing (NGS) is widespread and validated in many areas of research, although it is still limited in medical oncology [1,2,3,4,5,6,7].

The European Society for Medical Oncology (ESMO) Precision Medicine Working Group aimed to provide recommendations for the use of NGS in daily clinical practice. To do this, the most common mutations in the eight cancers with the highest global mortality rates were identified and classified using the ESMO Scale for Clinical Actionability of Molecular Targets (ESCAT). The ESCAT classification system determines the “actionability” of a mutation by assessing the level of evidence supporting the relationship between genomic alterations and the efficacy of targeted drugs. A lower number indicates stronger quantitative and qualitative evidence. To date, the ESMO recommends the routine clinical use of NGS in four types of cancer: advanced non-small-cell lung cancer, prostate cancer, ovarian cancer and cholangiocarcinoma. Considering the uncertain diagnostic value of NGS in other tumor histotypes, the ESMO acknowledges that oncologists and patients may choose to perform an NGS test. This will not incur additional costs for the public health system and the patient should be clearly informed about the likely poor test results. The ESMO recommends the off-label use of molecularly targeted drugs based on the result of a genetic test only in expanded access or compassionate use programs. Conversely, the ESMO encourages researchers to continue developing multigene sequencing techniques to enhance their effectiveness as screening tools. Finally, the ESMO currently does not recommend the use of the NGS test to determine the therapeutic course of patients with metastatic breast cancer (mBC) in daily clinical practice, for at least three reasons: sequencing for somatic pathogenic variants of *BRCA* cannot completely replace blood testing for germline mutations; less expensive techniques such as polymerase chain reaction can be used to determine mutational status of *PIK3CA*; and testing to identify HER2 amplification can be accurately performed by immunohistochemistry or in situ hybridization [8].

The goal of this retrospective study is to evaluate and quantify the usefulness of NGS testing in select target therapies. The aim is to implement precision medicine for metastatic breast cancer in daily clinical practice. For this purpose, the feasibility and clinical relevance of NGS tests on mBC samples of patients treated at the Modena Cancer Center were analyzed. These patients underwent NGS assays to determine the potential for molecular target therapy and to evaluate the prognostic implications of such treatment options.

## 2. Results

### 2.1. Population Characteristics

A total of 101 mBC patients treated at Modena Cancer Center between January 2015 and April 2022 were enrolled in this study. Median age at diagnosis was 50 years (range 29–76 years). Overall, 35.6% were de novo mBC and 83.1% of patients were alive at the end of the study.

A total of 77.2% of patients had ductal histotype. The most frequent phenotype was luminal A (51.5%), followed by luminal B (33.7%), HER2-positive (9.9%) and triple-negative (4.9%). Additionally, 35.6% of patients had “de novo” metastatic breast cancer at diagnosis. At the time of diagnosis of mBC, metastases affected more sites in 52.5% of patients, 14.8% had bone-only disease, and one patient had exclusive CNS localization. Overall, 67.3% of metastatic lesions had the same phenotype as the primary tumor. All patients received a median of 2 lines (range 0–14) of systemic treatment for metastatic disease, and 60.4% received chemotherapy in an early setting (Table 1).

### 2.2. Population Molecular Profile

All patients received NGS analysis on tumor tissue: 47 from primary cancer, 52 (51.5%) from metastatic sites (of which 7 were from bone, 8 from skin, 3 from lymph nodes, 21 from liver, 8 from lung and 5 from other tissues) and 2 from liquid biopsy. Overall, 40 patients were wild-type, while 61 patients had pathogenic mutations. Of these, 35 were carriers of a single mutation, 11 had two mutations and 15 had three or more mutations. Of these, 46 were tier I–II mutations according to ESCAT scale classification (Table 2).

A total of 85 mutations were found: the most frequently altered gene was *PIK3CA* (40/85, 47%), followed by *FGFR* (16/85, 18.8%), *ERBB2* (10/85, 11.7%), *MAP2K1* (6/85, 7%), *CDK* (4/85, 4.7%) and *ESR1* (3/85, 3.5%); other mutations included *ALK*, *JAK/STAT*, *MYC* and *BRAF/MEK* (6/85, 7%).

**Figure 1:**
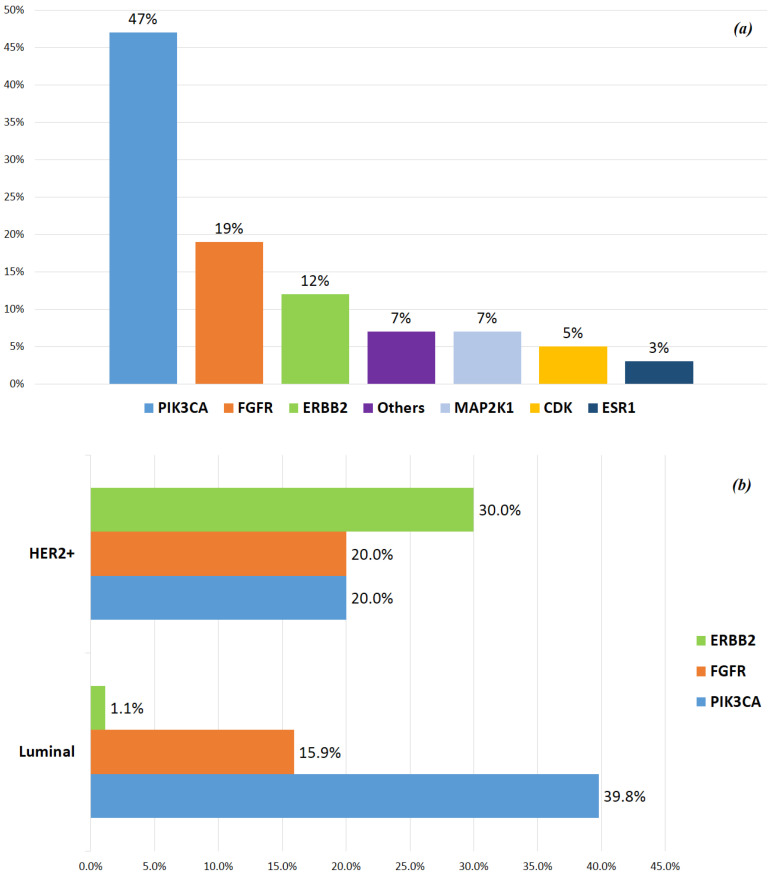
(**a**) Main genetic alteration incidence. (**b**) Distribution of the main mutations related to tumor phenotypes at diagnosis. #Triple-negative tumors were not considered in this figure, because only 2 of 5 patients showed genetic mutations (*CDK6* and *MYC*, respectively).

In patients with luminal-like phenotype at diagnosis, the most frequently mutated gene during metastatic disease was *PIK3CA* (39.8%); in those with HER2+ phenotype, *ERBB2* was the most frequently altered gene (30%) (Figure 1a,b).

### 2.3. Prognostic Impact of NGS-Based Analysis

**Figure 2:**
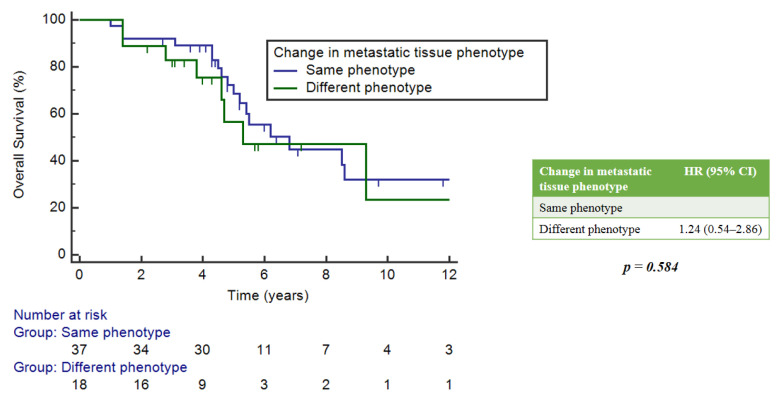
Correlation between changes in metastasis phenotype versus primary tumor and OS.

After 57 months of median follow-up (range 4–203), 99/101 pts were included in the final survival analysis. Of these, 53 patients underwent a biopsy at metastatic sites, 32.7% of whom showed a different phenotype from the primary tumor. However, there was no significant correlation between the phenotype change and OS when compared to patients who maintained the same phenotype as at initial diagnosis (*p* = 0.584) (Figure 2).

**Figure 3:**
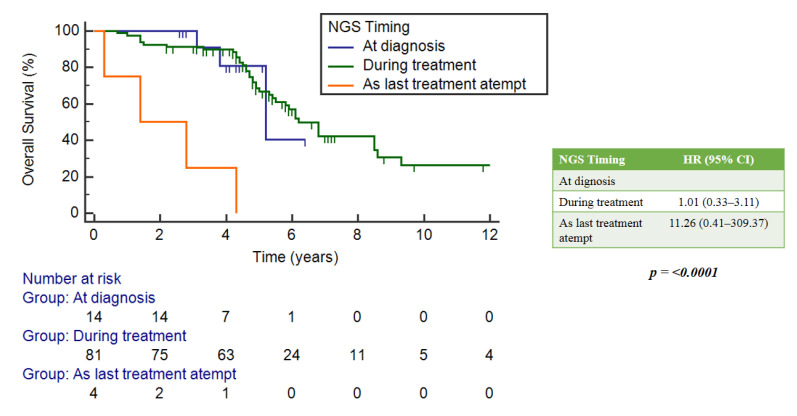
Correlation between OS and timing of NGS analysis.

There was no statistically significant implication between OS and the timing of NGS analysis, whether it was performed at diagnosis, during treatment or as a last therapeutic attempt. However, the latter group showed a numerically worse median OS (mOS) of 1.4 years (95%CI: 0.3–4.3) compared to 5.2 (5.2–5.2) and 6.2 (5.5–8.5) years for the other two groups, respectively (Figure 3).

**Figure 4:**
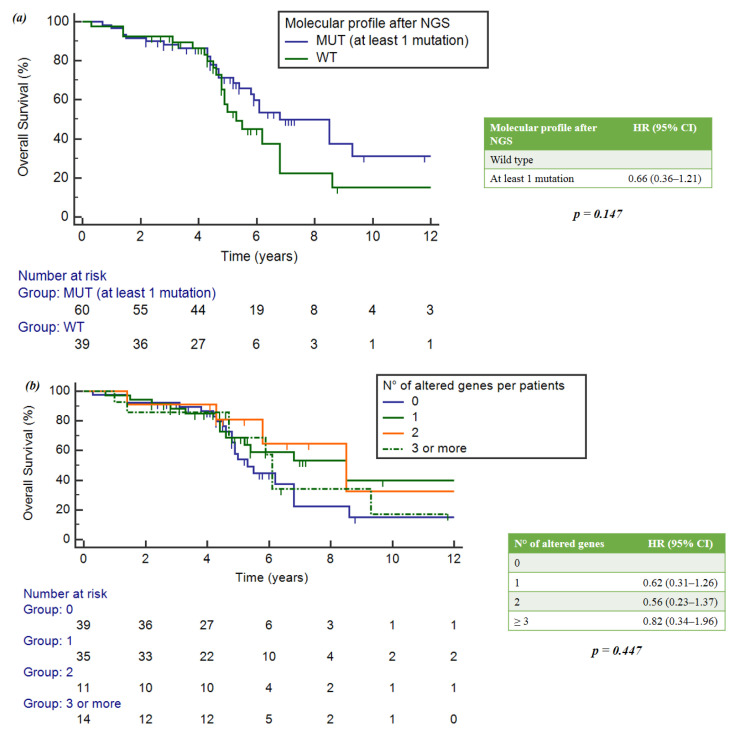
(**a**) Impact of gene alterations on OS. (**b**) Correlation between number of mutations per patient and OS.

No statistically significant differences in OS were observed between wild-type and mutated patients (*p* = 0.147) (Figure 4a).

When considering the number of mutations per patient, the group with single mutations had an mOS of 8.5 years (95%CI = 5.2–8.5), the group with two mutations showed an mOS of 8.5 years (5.8–16.9) and the group with three mutations showed an mOS of 6.1 years (4.7–9.3). Wild-type patients achieved a numerically lower mOS of 5.3 years (4.8-6.8). However, no statistically significant differences were observed (*p* = 0.447) (Figure 4b).

Forty mutations of *PIK3CA* were found. The mOS was 6.8 years (95%CI = 5.4–9.3) in the *PIK3CA*-mut population, and 6.1 years (5.0–8.6) in the *PIK3CA* wild-type group.

**Figure 5:**
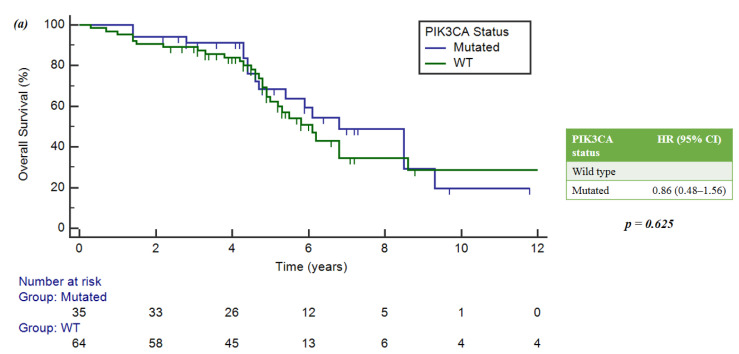

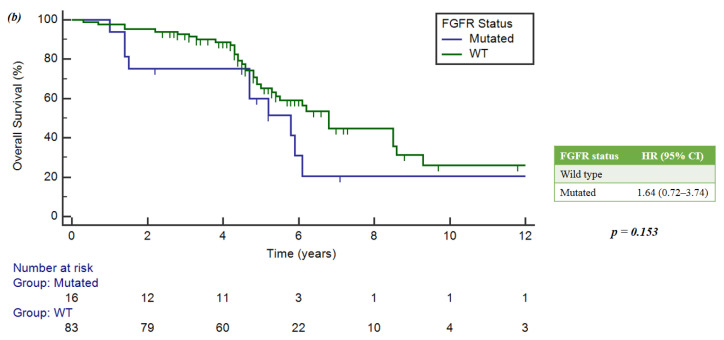
(**a**) Implication of *PIK3CA* mutations and OS. (**b**) Implication of *FGFR* mutations and OS.

In the FGF receptor family, the *FGFR*-mut group had an mOS of 5.8 years (4.7–6.1), while the *FGFR* wild-type group had a median survival of 6.8 years (5.4–8.6). In both cases, these correlations were not statistically significant: *p* = 0.625 and *p* = 0.153, respectively (Figure 5a,b).

### 2.4. Role of the NGS Test in the Population Treated with CDK4/6 Inhibitors

**Figure 6:**
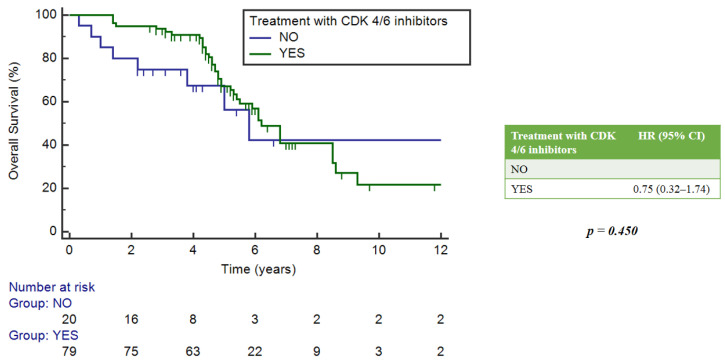
Impact of NGS on overall survival in patients who received CDK4/6 inhibitors.

The impact of NGS assays was estimated in the subgroup of patients treated with CDK4/6 inhibitors, which constituted 78% of the study population. Among them, 39.3% had no mutation and 60.7% had at least one mutation. There were no statistically significant differences in mOS between these two groups of patients, with respective values of 6.2 years (95%CI = 4.9–6.8) and 6.8 years (5.4–9.3), *p* = 0.403 (Figure 6).

### 2.5. Actionable Mutations

**Figure 7:**
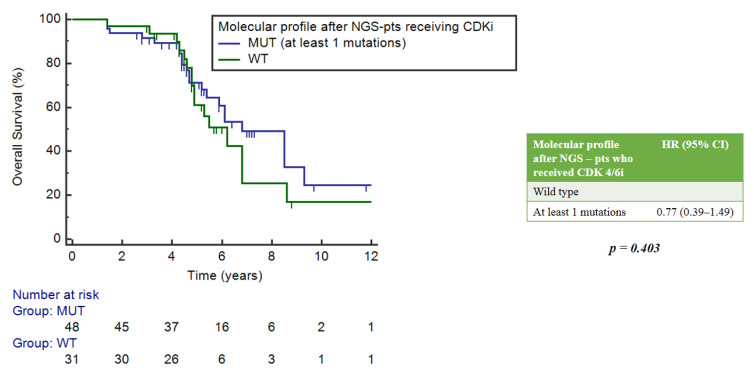
Correlation between gene mutations according to ESCAT scale and OS.

Out of the 61 patients with pathogenic mutations, 35 had at least one actionable tier I mutation according to the ESCAT scale, for which the use of the specific drug is recommended. The most frequent actionable tier I mutation was *PIK3CA* (47%). Patients with tier I–II mutations had an mOS of 8.5 years (95%CI = 5.9–9.3), while patients with tier ≥ 3 mutations had an mOS of 5.2 years (3.3–16.9). There were no statistically significant differences in OS observed between these groups (*p* = 0.549) (Figure 7).

Overall, 9% (9/101) of patients received further treatment with a targeted drug based on the their mutational profile as determined by NGS analysis. All patients had a luminal A-tumor and had largely been pretreated for metastatic disease, with a median of 5 treatment lines [range 2–14]. Overall, 89% of patients had *PIK3CA* mutations and received targeted therapy with alpelisib plus fulvestrant. Although mutations in the *FGFR* family genes are not currently classified as actionable level I or level II mutations according to the ESCAT, a patient with *FGFR1* amplification received TAS120 after randomization in a clinical trial. At the end of the study, 44% of these patients were still alive. The median progression-free survival (PFS) of this sub-population was 3.8 months (Table 3).

## 3. Discussion

This real-world study demonstrates the usefulness of NGS analysis in detecting genetic alterations and cancer-related biomarkers. This allows for specific targeted agent therapy in approximately 10% of mBC patients tested.

In a previous analysis, we evaluated a panel of 25 genes involved in the mechanisms of targeted treatment resistance in 16 primary breast cancers and their matched recurrences. We observed that almost all patients had at least one alteration that could be targeted with approved or investigational therapeutics. This suggests that routine genomic profiling may be instrumental in individualized pathway-directed therapies. We also found substantial discordances between primary tumors and metastases, highlighting the need for analysis of metastatic tissue at recurrences, which may contribute to treatment selection [9]. The ESR1 mutation, a mechanism of acquired endocrine resistance in breast cancer, is an example. It is known that this mutation is rare in primary tumors (0–4%), but frequent in endocrine-resistant metastatic lesions (6–50%) [10]. Identifying the ESR1 mutation can change the therapeutic approach, helping to choose the most appropriate endocrine treatment (i.e., oral SERDs).

On the other hand, this study demonstrates that the use of targeted drug therapies is not always a direct result of the performance of the NGS test. This is due to variables such as the patient’s clinical condition, drug unavailability, limited access to clinical trials and the financial burden on the national health system.

Several studies have reported NGS test results in daily clinical practice [11,12,13], but the advantages of NGS in improving PFS and OS remain controversial. Few randomized trials have reported the use of NGS-based therapeutic approaches. SHIVA was a randomized phase 2 controlled precision medicine study which showed that the use of NGS did not improve the outcome, regardless of the type of cancer [14]. Other studies [15] have shown an improved response rate and PFS. The Safir02-BREAST [16] trial enrolled 1462 patients with pretreated HER2-negative metastatic breast cancer. Each patient underwent NGS analysis to assess the presence of druggable mutations. Patients who achieved a complete/partial response or a stable disease after six to eight courses of chemotherapy (CT) were randomized to receive either target therapy matched to genomics vs. maintenance CT (if they had a druggable mutation) or durvalumab vs. maintenance CT (in the absence of mutations). The study showed that the use of “target therapies matched to genomic” improved PFS (adjusted HR 0.41, 90%CI: 0.27–0.61, *p* < 0.001) in patients with mutations classified as level I-II according to the ESCAT. No improvement in PFS was observed for mutations classified above level II (HR 1.15, 95%CI 0.76–1.75) or for mutations unselected according to the ESCAT (HR 0.77, 95%CI 0.56–1.06, *p* = 0.109).

In our retrospective study, patients with metastatic breast cancer who received targeted treatment had a median PFS of 3.8 months. No comparative analysis was performed to assess the effectiveness of target versus non-target treatments, due to the heterogeneity of breast cancer subtypes and their treatments, according to current guidelines. However, our findings suggest that the use of the NGS test could benefit patients in daily clinical practice. This is because it could allow access to off-label use of targeted agents, especially considering the high number of level II mutations found in the mBC population [8].

This study presents some critical points. It was a retrospective and monocentric study, with a limited sample size of patients. Different NGS panels were used, based on the Institution’s availability over time. These limitations could potentially bias the study of genomic profiles in breast cancer. Despite these aspects, this study aimed to estimate the impact of NGS testing in daily clinical practice and to determine new targeted therapeutic choices.

## 4. Methods

This is a retrospective and monocentric study. NGS data were collected from 101 metastatic breast cancer patients treated at the Modena Cancer Center between January 2015 and April 2022. An NGS study on cancer tissue was performed at the Molecular Pathology Laboratory of the University Hospital of Modena. The identified mutations were classified to identify actionable and bootable molecular target treatments.

### 4.1. Ethical Committee

The study was approved by the Ethical Committee of the Area Vasta Emilia Nord (approval code 168/2021, approval date 28 September 2022). This observational research was reported according to STROBE guidelines (https://www.strobe-statement.org/ accessed on 5 January 2024), while the checklists are reported in the Supplementary Materials. The study was conducted according to the principles of good clinical practice, in accordance with the Helsinki Declaration on clinical trials. Data were analyzed in aggregate and anonymous form.

### 4.2. Patients

Patients eligible for the study had to meet the following inclusion criteria: age greater than or equal to 18 years; diagnosis of metastatic breast cancer; electronic medical record available at the Modena Cancer Centre; and NGS test on primary or metastatic breast cancer tissue performed as for clinical practice.

Clinical–pathological data included patient demographics; date of first diagnosis and recurrence; tumor characteristics; type and duration of previous neo/adjuvant chemotherapy and/or adjuvant hormone therapy if given; site of metastatic disease; type of biopsy (metastasis vs. primary); type and duration of treatments for metastatic disease; and patient status.

NGS data included NGS test run date; location of sampling (primary tumor, metastasis or liquid biopsy); gene panels used; gene variants identified; and effect of NGS results on therapeutic choice.

### 4.3. NGS Test

In all cases, DNA analysis was performed in the main cancer-related genes currently considered clinically significant: SNV (single-nucleotide variant); MNV (multi-nucleotide variant); and INDELs (short insertions and deletions). In more recent cases, NGS panels could be used to simultaneously analyze DNA and RNA, so that in addition to the above determinations, fusion transcripts such as NTRK1, NTRK2 and NTRK3 were also investigated.

NGS analysis was performed on nucleic acids extracted from formalin-fixed, paraffin-embedded biopsy tissue and/or surgical specimens. Manual microdissection of the neoplastic area was performed from 4–6 sections with 10 μm of thickness. DNA and RNA extraction was performed using the automatic magcore extractor Super (RBC Bioscience, New Taipei City, Taiwan), using the magcore kit Genomic DNA FFPE One-Step Kit and magcore Total RNA FFPE One-Step Kit (RBC Bioscience). The resulting nucleic acids were then quantified by a Qubit fluorometer (Invitrogen, Waltham, MA, USA) and diluted to the specific concentration required by the NGS panel used.

The technologies used in our study were the Illumina MiSeq platform and the Ion Torrent Ion-S5 with Ion-Chef system.

The gene panels used for the MiSeq Illumina platform were as follows:

(1) Myriapod NGS-IL 56G Onco Panel (Diatech Pharmacogenetics, Iesi, Italy): allows the analysis of hotspot variants on DNA in 56 cancer-related genes (*ABL1, AKT1, ALK, APC, ATM, BRAF, CDH1, CDKN2A, CSF1R, CTNNB1, DDR2, DNMT3A, EGFR, ERBB2, ERBB4, EZH2, FBXW7, FGFR1, FGFR2, FGFR3, FLT3, FOXL2, GNA11, GNAQ, GNAS, HNF1A, HRAS, IDH1, IDH2, JAK2, JAK3, KDR, KIT, KRAS, MA2K1, MET, MLH1, MLP, MSH6, NOTCH, NPM1, NRAS, PDGFRA, PIK3CA, PTEN, PTPN11, RB1, RET, SKT11, SMAD4, SMARCB1, SMO, SRC, TP53, TSC1, VHL*). It requires three days of manual work and then loading on the platform.
(2) Myriapod NGS Cancer Panel DNA (Diatech Pharmacogenetics): includes DNA analysis of 16 genes with current clinical utility (*ALK, BRAF, EGFR, ERBB2, FGFR3, HRAS, IDH1, IDH2, KIT, KRAS, MET, NRAS, PDFRA, PIK3CA, RET, ROS1*). It requires 1–2 days of manual work, followed by loading on the platform.

The Oncomine Dx Targer Test (ThermoFisher Scientific, Waltham, MA, USA) panel was used for the Ion Torrent platform. Introduced in March 2020, it allows the simultaneous evaluation of somatic alterations in DNA and RNA. In detail, it includes the following:

(1) DNA analysis of the hotspot variants (SNV, MNV, INDELs) of clinical relevance for 41 genes (*AKT1, ALK, AR, BRAF, CCND1, CDK4, CDK6, CTNNB1, DDR2, EGFR, ERBB2, ERBB3, ERBB4, ESR1, FGFR1, FGFR2, FGFR3, FGFR4, GNA11, GNAQ, HRAS, IDH1, IDH2, JAK1, JAK2, JAK3, KIT, KRAS, MAP2K1, MAP2K2, MET, MTOR, MYC, MYCN, NRAS, PDGFRA, PIK3CA, RAF1, RET, ROS1, SMO*);
(2) RNA analysis of 23 gene fusion transcripts (*ABL1, ALK, AKT3, AXL, BRAF, EGFR, ERBB2, ERG, ETV1, ETV4, ETV5, FGFR1, FGFR2, FGFR3, MET, NTRK1, NTRK2, NTRK3, PDGFRA, PPARG, RAF1, RET, ROS1*).

### 4.4. Bioinformatics Analysis

The panels described above, all with CE-IVD marking and inserted in the context of health care activity, include automated analysis processes and dedicated analysis software.

Regardless of the panel and platform used, the NGS process generates raw sequencing data (reads) that must undergo complex bioinformatic analysis procedures. Schematically, this is divided into the following procedures:

Primary analysis: performed directly within the sequencing instrument, this consists of the conversion of the measured signal (fluorescence in the case of Illumina platforms, pH change in the IonTorrent platform) in nucleotide sequence (base calling);

Secondary analysis: foresees the alignment of the generated reads to the reference genome and the call of the variants (variant calling), the identification of the altered bases relative to the deposited wild-type sequence;

Tertiary analysis: consists of filtering and prioritizing the identified variants according to the initial diagnostic question and involves the consultation of genomic databases that collect the gene variants, allowing the characterization of the expected pathogenic effect and the level of clinical and therapeutic significance.

Secondary analysis tools used for variant calling were Myriapod NGS Data Analysis Software (Diatech Pharmacogenetics) for the Myriapod Panels and Ion Reporter Software (Thermofisher, Waltham, MA, USA) for the Oncomine panel. The next steps of biological characterization, prediction of pathogenicity and classification of the variant involved the use of different bioinformatic tools, including Varsome, Franklin, OncoKb, MyCancerGenome, cBioPortal, etc. Variant Classification was made according to ACMG, AMP/ASCO/CAP and ESCAT guidelines [8,17,18].

### 4.5. Statistical Analysis

The data collected were summarized by descriptive statistical analysis, calculating frequencies and percentages for qualitative variables and medians for continuous variables. The NGS gene mutations found in cancer tissue samples were reported. Concerning major gene mutations, the frequency of detection was calculated and correlated with survival. Actionable mutations were identified according to ESCAT classification, as for ESMO guidelines [19,20]. The overall survival (OS) was calculated as the time interval from diagnosis of metastatic disease to the date of the last observation or death for any cause. Progression-free survival (PFS) was assessed as the time interval between the start of the target therapy and its discontinuation due to patient death or any other reason. The statistical program used to analyze the results was MedCalc.

## 5. Conclusions

In conclusion, based on ESMO recommendations [8], the routine use of Next-Generation Sequencing panels cannot be recommended for patients with advanced breast cancer at the moment. However, its use in clinical trials remains desirable, when available.

Despite the small sample size and limited follow-up, this study demonstrates how NGS analysis results can provide information to guide therapeutic choices towards target drugs in a non-negligible percentage of patients. These represented approximately 10% of the total study population. Unfortunately, due to the limited sample size and the heterogeneity of the population and treatments received, it was not possible to assess the impact on patient survival.

These results are in line with other research [21,22,23]. It is likely that NGS analysis will become increasingly available in clinical practice, due to increased accessibility to such technologies, reduced execution costs, and the emergence of an increasing number of molecular target drugs. It is also desirable that all this should include the presence of an appropriate Molecular Tumor Board (MTB).

## Figures and Tables

**Table 1:** Population and disease characteristics.

	N = 101	(%)
Median Age at diagnosis		
	52 years	[29–82 years]
Stage at diagnosis		
I	9	(8.9%)
II	32	(31.7%)
III	24	(23.8%)
IV	36	(35.6%)
Tumor phenotype at diagnosis		
Luminal A-like	52	(51.5%)
Luminal B-like	34	(33.7%)
HER2-positive	10	(9.9%)
Triple-negative	5	(4.9%)
N° of metastatic sites at diagnosis of mBC		
1	48	(47.5%)
2	46	(45.5%)
≥3	7	(7%)
Type of metastatic sites at diagnosis of mBC		
Bone only	15	(14.8%)
Visceral only	32	(31.7%)
CNS only	1	(1%)
Bone and visceral	53	(52.5%)
Concordance in tumor phenotype between metastases and primary * (N = 52 biopsies on metastatic site)		
Same tumor phenotype	35	(67.3%)
Change in tumor phenotype	17	(32.7%)
Prior (neo)adjuvant treatments		
Chemotherapy	61	(60.4%)
Endocrine therapy	59	(58.4%)
Systemic treatments for metastatic disease (N° of lines)		
1	31	(30.7%)
2	19	(18.8%)
≥3	51	(50.5%)

**Table 2:** Population molecular profile.

	N = 101	(%)
Origin of tumor tissue tested for NGS		
Primary tumor	47	(46.5%)
Metastatic site	52	(51.5%)
− Bone	7	(7%)
− Skin	8	(7.9%)
− Lymph nodes	3	(3%)
− Liver	21	(20.8%)
− Lung	8	(7.9%)
− Other tissues	5	(5%)
Liquid biopsy	2	(2%)
N° of mutations per patients		
Wild type	40	(39.6%)
Mutated	61	(60.4%)
− 1 mutation	35	(34.7%)
− 2 mutation	11	(10.9%)
− ≥3 mutations	15	(14.9%)
Mutation classification according to ESCAT scale		
− Tier I	35	(57.4%)
− Tier II	11	(18.0%)
− Tier > II	15	(24.6%)

**Table 3:** Characteristics of patients treated with target agents after NGS profiling.

Patients	N° Prior Systemic Treatments For Mbc	Mutations	Target Treatment	PFS (Months)	State of Patient
1	14	*PIK3CA*	Alpelisib + Fulvestrant	3.8	Dead
2	9	*PIK3CA*	Alpelisib + Fulvestrant	0.5	Dead
3	2	*PIK3CA*	Alpelisib + Fulvestrant	1.6	Alive
4	2	*PIK3CA*	Alpelisib + Fulvestrant	24.2	Alive
5	5	*PIK3CA*	Alpelisib + Fulvestrant	13.1	Dead
6	6	*PIK3CA*	Alpelisib + Fulvestrant	8.5	Dead
7	3	*PIK3CA*	Alpelisib + Fulvestrant	1.0	Alive
8	6	*PIK3CA*	Alpelisib + Fulvestrant	2.1	Alive
9	3	*FGFR1/2*	TAS-120	11.7	Dead

## Data Availability

The datasets presented in this article are not readily available due to privacy and technical limitations. Requests to access the datasets should be directed to corresponding author.

## Supplementary Materials

The following supporting information can be downloaded at: https://www.mdpi.com/article/10.3390/ijms25052490/s1.
